# Quantitative Spinal Cord Deformation Is Associated with Neurologic Status in High-Grade Thoracic Metastatic Epidural Spinal Cord Compression: A Retrospective Surgical Cohort

**DOI:** 10.3390/jcm15197702

**Published:** 2026-10-05

**Authors:** Ilhan Yilmaz, Baran Taskala, Muhammed Bayindir, Aydin Talat Baydar

**Affiliations:** Department of Neurosurgery, Basaksehir Cam and Sakura City Hospital, 34480 Istanbul, Türkiye; baran.taskala@saglik.gov.tr (B.T.); muhammed.bayindir1@saglik.gov.tr (M.B.); aydintalat.baydar@saglik.gov.tr (A.T.B.)

**Keywords:** metastatic epidural spinal cord compression, spinal metastasis, Bilsky grade, Frankel grade, magnetic resonance imaging, spinal cord deformation, cord compression ratio

## Abstract

**Background/Objectives:** Neurologic presentation varies among patients with high-grade metastatic epidural spinal cord compression (MESCC), even within Bilsky grades 2–3. We examined whether quantitative preservation of spinal cord caliber is associated with neurologic intactness at presentation. **Methods:** We retrospectively studied 73 adults who underwent surgery for thoracic Bilsky grade 2–3 MESCC between June 2020 and December 2025. Preoperative neurologic status was classified as Frankel E versus Frankel A-D. Blinded observers measured anteroposterior and transverse cord diameters at maximal compression and at the first tumor-free cranial and caudal pedicle levels. A normalized two-adjacent cord compression ratio (CCR) was available in 71 patients. Modified Poisson regression estimated prevalence ratios for Frankel-E status, adjusted for Bilsky grade; logistic, Firth, and cranial-reference analyses were used as sensitivity analyses. **Results:** Twenty-two patients (30.1%) were Frankel E. Two-adjacent CCR was higher in Frankel-E than Frankel A-D patients (median 0.521 vs. 0.439; *p* = 0.013). Each 0.10 increase in CCR was associated with greater prevalence of Frankel-E status after Bilsky adjustment (PR 1.23, 95% CI 1.04–1.47; *p* = 0.018). Logistic, Firth, and cranial-reference analyses were generally consistent in direction; the solid-tumor-only estimate also remained positive but was less precise and included the null. Interobserver agreement for CCR was acceptable but imprecise (ICC[A,1] = 0.758, 95% CI 0.433–0.911). **Conclusions**: In this surgically selected cohort, normalized cord-caliber preservation was associated with neurologic intactness after accounting for Bilsky grade. CCR should be regarded as a research phenotype rather than an individual-patient predictor or treatment threshold, pending prospective validation in broader surgical and nonsurgical populations.

## 1. Introduction

Metastatic epidural spinal cord compression (MESCC) is one of the most consequential complications of spinal metastatic disease. Loss of motor function can compromise ambulation, independence, rehabilitation, and the ability to proceed with systemic oncologic treatment [1]. Direct decompressive surgery has an established role in appropriately selected patients [2], but contemporary management is broader than a surgery-versus-radiotherapy decision and integrates neurologic function, tumor biology and radiosensitivity, mechanical stability, systemic condition, and expected survival [3,4,5]. Randomized evidence and current practice guidance support an expanding role for spine stereotactic body radiotherapy in selected patients [6,7], while recent reviews emphasize hybrid separation surgery and less invasive operative strategies as components of multidisciplinary care [8,9,10,11].

The Bilsky epidural spinal cord compression (ESCC) scale provides a practical and reproducible description of epidural disease on axial T2-weighted magnetic resonance imaging (MRI) [12]. Grade 2 denotes spinal cord compression with residual cerebrospinal fluid visible around the cord, whereas grade 3 denotes compression with complete effacement of the surrounding cerebrospinal fluid. Both are considered high-grade disease and are embedded in modern spine-oncology decision frameworks [3,4].

Clinical function, however, does not map perfectly onto the ESCC category. Patients with apparently similar high-grade thoracic compression can present with markedly different neurologic examinations. Uei et al. showed a relationship between ESCC severity and paralysis, but also demonstrated clinical heterogeneity within radiographic grades [13]. Klein et al. found that pathologic fracture alone did not explain severe paralysis in a surgical MESCC cohort [14]. Similar variability has been reported in MRI-based studies of treatment-related ambulatory function [15,16,17,18].

A limitation of the Bilsky scale is inherent to its purpose. It describes the relationship among epidural tumor, cerebrospinal fluid, and the cord, but it does not directly quantify how much the cord itself has been deformed. Two patients can both meet the definition of Bilsky grade 3 while retaining different residual cord calibers. Kim et al. addressed this problem by measuring anteroposterior (AP) and transverse cord diameters and normalizing their product to adjacent noncompressed cord; their cord compression ratio (CCR) was associated with postoperative ambulatory function in surgically treated symptomatic MESCC [17].

The present study addresses a different question. Rather than asking whether an imaging measurement predicts postoperative ambulation, we asked whether quantitative cord deformation helps explain why some patients remain neurologically intact despite Bilsky grade 2 or 3 thoracic MESCC. We re-evaluated a surgically treated high-grade thoracic cohort using normalized CCR and detailed MRI phenotyping. The primary objective was to determine whether preservation of normalized cord caliber was associated with Frankel-E status after accounting for Bilsky grade. We also evaluated measurement reproducibility and, as a secondary longitudinal analysis, examined early postoperative neurologic recovery among patients who presented with an objective deficit.

This was not designed as a prediction-model study. We did not derive a cutoff, optimize a receiver-operating-characteristic threshold, or propose a treatment rule. The primary analysis is a cross-sectional association between preoperative imaging and neurologic status within a selected surgical population.

## 2. Materials and Methods

### 2.1. Study Design, Setting, and Patient Selection

This retrospective observational study was conducted at Basaksehir Cam and Sakura City Hospital, Istanbul, Türkiye, a tertiary referral center for neurosurgery and spine oncology. Consecutive adults who underwent surgery for spinal metastatic disease between June 2020 and December 2025 were screened. The institutional review board approved the study (Approval No. 31; approval date 27 January 2026). The requirement for individual informed consent was waived because the analysis used retrospective, anonymized clinical data. The study was conducted in accordance with the Declaration of Helsinki and is reported in accordance with the STROBE statement [19].

The source population comprised 143 consecutive surgically treated patients with spinal metastatic disease. Seventy patients were excluded: 54 because the index lesion was nonthoracic, 5 because the metastatic disease was intradural, 8 because the epidural component was Bilsky grade 1c rather than grade 2 or 3, and 3 because preoperative MRI was unavailable. The final cohort comprised 73 patients with a thoracic index lesion and preoperative Bilsky grade 2 or 3 MESCC (Figure 1). No additional patient was excluded for laboratory data, missing systemic variables, or a secondary imaging variable.

When disease involved more than one vertebral level, the index lesion was defined as the level producing the greatest epidural spinal cord compression and/or the principal level targeted during surgical decompression. All 73 final-cohort patients had preoperative MRI adequate for Bilsky grading and measurement at the maximal-compression level. In two patients, a reliable caudal reference level could not be obtained because of caudal transition of the spinal cord; these patients remained in the cohort and contributed to the full-cohort cranial-reference sensitivity analysis rather than being excluded. The primary two-adjacent CCR was therefore available in 71 patients.

Because every patient underwent surgery, the cohort should be regarded as a selected surgical referral population rather than an epidemiologic sample of all patients with high-grade thoracic MESCC. Indications for surgery were individualized in routine multidisciplinary care and could include neurologic compromise, mechanical instability or pain, high-grade epidural disease, tumor biology, and the broader oncologic treatment plan.

During preparation of this manuscript, the authors used ChatGPT (v5.6 Sol, OpenAI, San Francisco, CA, USA) for language and structural refinement after completion of the data analysis. The authors reviewed and edited the output and take full responsibility for the content of the publication.

### 2.2. Neurologic and Clinical Assessment

Neurologic status was classified using the Frankel grading system. The preoperative grade was taken from the earliest specialist neurologic examination corresponding to the index surgical admission. For the primary analysis, Frankel E was considered neurologically intact, whereas Frankel A-D represented the presence of an objective neurologic deficit. This dichotomy was chosen to address complete neurologic preservation and was not intended to imply equivalent severity among Frankel grades A through D. Frankel grading was used because it was consistently documented at both the preoperative and early postoperative time points throughout the retrospective study period. Complete standardized International Standards for Neurological Classification of Spinal Cord Injury (ISNCSCI) examinations, including the motor, sensory, and sacral components required for American Spinal Injury Association Impairment Scale (AIS) classification, were not routinely recorded. Retrospective conversion from the available documentation to AIS grades was therefore considered unreliable.

For descriptive postoperative transition analyses, Frankel D–E was treated as an ambulatory-capable neurologic category and Frankel A–C as a nonambulatory-level category. This classification was used as a Frankel-based neurologic surrogate; directly observed walking status was not analyzed as a separate endpoint. Postoperative neurologic status was assessed on postoperative day 10–14. Improvement was defined as an increase of at least one Frankel grade and deterioration as a decrease of at least one grade. The postoperative day 10–14 examination was selected to characterize early neurologic change during the index recovery period; it was not intended to represent durable functional outcome.

Among patients who presented with an objective deficit, deficit duration was defined as the interval between the first patient-reported or physician-documented motor weakness, gait failure, or loss of ambulation and surgery. If the clinical record did not permit a reliable onset time, duration was left missing rather than retrospectively inferred.

Bilsky grade was assessed on preoperative MRI according to the original ESCC classification [12]. For quality assurance, 30 preoperative MRI studies were randomly selected and independently graded by two spine-oncology neurosurgeons blinded to clinical outcome data. Exact agreement was 27 of 30 examinations, with a weighted Cohen kappa of 0.734.

Mechanical stability was characterized using the Spinal Instability Neoplastic Score (SINS) [4]. For the final dataset, SINS was calculated from the individual score components and this total was used in all analyses. Pathologic fracture and kyphosis were cross-checked against the corresponding SINS components. Mechanical variables were treated primarily as descriptors because instability contributes to the decision to operate and is therefore strongly affected by surgical selection.

### 2.3. MRI Review and Quantitative Measurement Protocol

Preoperative MRI examinations were reviewed on the institutional picture archiving and communication system. Imaging had been obtained during routine clinical care over several years; scanner manufacturer and acquisition parameters were therefore not completely uniform. The principal quantitative measures were normalized within each patient rather than interpreted as absolute cord dimensions, reducing but not eliminating the effect of platform and acquisition heterogeneity [20].

Axial T2-weighted images formed the basis of the quantitative analysis. Sagittal images were reviewed simultaneously to confirm the vertebral level, the longitudinal extent of epidural disease, and the location of selected axial slices. Observers performing quantitative measurements were blinded to Frankel grades.

The axial T2 series was reviewed sequentially through the compressed segment. The maximal-compression slice was defined as the axial image showing the greatest visible deformation of the spinal cord by the epidural lesion. When compression extended across more than one vertebral level, all involved levels were reviewed and the single slice with greatest cord deformation was selected. The number of levels demonstrating direct cord compression was recorded separately to preserve information about longitudinal disease burden.

Reference measurements were not taken from an arbitrary immediately adjacent image. Beginning at the compressed segment, the observer reviewed the MRI cranially and caudally until reaching the first neighboring pedicle level at which no epidural mass was visible. These levels served as the cranial and caudal references. The pedicle provided a reproducible vertebral landmark, while the absence of visible epidural tumor was intended to avoid normalizing the compressed cord to another obviously involved level. A valid cranial reference was available in all 73 patients. A reliable caudal spinal cord reference was unavailable in two patients.

At maximal compression and at each available reference level, AP and transverse cord diameters were measured in millimeters using electronic PACS calipers. The AP diameter was placed between the visible ventral and dorsal cord margins, and the transverse diameter was measured orthogonally between the lateral margins on the same image. Measurements were confined to the spinal cord and excluded cerebrospinal fluid, dura, epidural tumor, and osseous structures.

The product of AP and transverse diameter was used as a simple two-dimensional area surrogate: A = AP × T. Because the compressed spinal cord is not necessarily rectangular, elliptical, or geometrically regular, this product was not interpreted as a true segmented cross-sectional area.

The primary quantitative measure was the two-adjacent cord compression ratio (CCR), adapted from the normalization concept described by Kim et al. [17]: CCR2adj = Amax/[(Acranial + Acaudal)/2], where Amax is the AP × transverse area surrogate at maximal compression. Values closer to 1 denote greater preservation of cord caliber relative to the patient’s own adjacent cord; lower values indicate greater deformation. The two-adjacent CCR was available in 71 of 73 patients. A full-cohort cranial-reference ratio, CCRcranial = Amax/Acranial, was also calculated and served as a sensitivity analysis. Neither a previously published cutoff nor a newly optimized threshold was used.

Absolute cranial and caudal reference area surrogates were compared between neurologic groups to determine whether a difference in normalized CCR might simply reflect constitutionally different baseline cord size. The area ratio was also decomposed into AP and transverse preservation ratios by dividing the compressed AP or transverse diameter by the mean corresponding diameter at the two reference levels. These dimensional ratios were treated as secondary descriptions of cord shape rather than competing primary exposures. The measurement workflow and representative Bilsky grade 3 examples with differing quantitative cord deformation and neurologic status are illustrated in Figure 2.

Additional MRI variables included intramedullary T2 hyperintensity, circumferential extent of compression, dominant compression direction, and the number of directly compressed levels. T2 hyperintensity was recorded when abnormal high signal was visible within the cord at or adjacent to maximal compression. Circumferential involvement was categorized as 0–90 degrees, 90–180 degrees, 180–270 degrees, or 270–360 degrees. Dominant compression was classified as ventral, ventrolateral, posterolateral, posterior, or circumferential.

### 2.4. Interobserver Reliability

Fifteen patients were randomly selected from the final cohort for complete independent repeat assessment by a second observer. The second observer was blinded to Frankel grade and independently reviewed the MRI, selected the maximal-compression slice, and repeated all measurements using the same pedicle-based reference rule. The reliability experiment therefore incorporated both slice selection and caliper placement, rather than testing caliper placement on a slice predetermined by the first observer.

Agreement was evaluated using a two-way, absolute-agreement, single-measure intraclass correlation coefficient, reported as ICC(A,1) with 95% confidence intervals [21]. Individual AP and transverse measurements and derived ratios were assessed. For the primary two-adjacent CCR, agreement was additionally examined using Bland–Altman analysis [22]. Bias was defined as Observer 2 minus Observer 1, and the 95% limits of agreement were calculated as the mean difference ±1.96 standard deviations. Pearson and Spearman correlations were calculated descriptively but were not used as substitutes for agreement statistics. Complete reliability statistics for individual dimensions and derived ratios are provided in Supplementary Table S2.

### 2.5. Oncologic, Systemic, and Operative Variables

Clinical and oncologic variables were abstracted from the established study database. Primary tumor groups were categorized for descriptive purposes as lung, multiple myeloma, breast, gastrointestinal, prostate, renal cell carcinoma, lymphoma, and other solid tumors. Hematologic malignancies comprised multiple myeloma and lymphoma. Visceral metastasis was defined as documented extraskeletal visceral metastatic disease before the index operation. The complete demographic, oncologic, systemic, mechanical, imaging, and operative profile is provided in Supplementary Table S1. Preoperative treatment exposure was characterized as prior radiotherapy to any spinal site, prior radiotherapy to the index level, and prior systemic anticancer therapy. Treatment timing relative to surgery was retained where available. Corticosteroid exposure before MRI could not be reconstructed consistently with respect to dose and timing across the study period and was therefore not analyzed as a formal covariate.

ASA physical status and ECOG performance status were retained for descriptive characterization but were not entered into the primary neurologic model. Both were assessed during the same clinical episode as the neurologic examination, and ECOG in particular is directly influenced by mobility and independence. With the modest number of Frankel-E patients, including such temporally overlapping measures would also have risked overadjustment and model instability.

The hemoglobin-albumin-lymphocyte-platelet (HALP) index was retained only as an exploratory systemic-context variable in Supplementary Table S4. It was not used for eligibility, was not a primary explanatory biomarker, and did not enter the principal neurologic model. Missing laboratory values therefore did not cause exclusion from the cohort.

Surgical strategy was individualized according to neurologic examination, mechanical stability, epidural disease, tumor biology, systemic condition, and anticipated oncologic treatment. The present study was not designed to compare operative techniques. Operative category was not entered into the baseline neurologic model because treatment choice occurred downstream of the presentation phenotype.

### 2.6. Statistical Analysis

Statistical analyses were performed in R version 4.5.2. Continuous variables are reported as median and interquartile range (IQR), with mean and standard deviation additionally provided for selected quantitative MRI measures. Categorical variables are presented as number and percentage. Unadjusted group comparisons used the Wilcoxon rank-sum test for continuous variables and Fisher’s exact test for categorical variables when appropriate.

The primary outcome was Frankel E versus Frankel A–D at presentation. Because Frankel-E status occurred in approximately 30% of the cohort, the principal effect measure was a prevalence ratio rather than an odds ratio. Modified Poisson regression with a log link and HC3 heteroscedasticity-consistent robust covariance estimation was used to calculate prevalence ratios (PRs) and 95% confidence intervals [23,24]. CCR was scaled so that its coefficient represented a 0.10 increase in the ratio. Modified Poisson models were fitted with stats::glm() using a Poisson family and log link; HC3 heteroscedasticity-consistent covariance matrices were obtained with sandwich::vcovHC(type = “HC3”) and coefficient tests with lmtest::coeftest(). Firth logistic sensitivity analyses used logistf::logistf(), and ICC estimates used irr::icc() with a two-way absolute-agreement, single-measure specification.

The primary model was intentionally restricted to two variables: two-adjacent CCR and Bilsky grade. This model was selected on conceptual grounds before the final locked inferential analysis and was not derived by significance-based or stepwise variable selection. CCR represented the principal quantitative imaging exposure, while Bilsky grade was retained as the established categorical measure of epidural compression severity against which the quantitative metric was being evaluated. With 21 Frankel-E patients contributing to the two-adjacent analysis, broader adjustment for every observed baseline difference would have increased the risk of overfitting and unstable estimates. Other potentially relevant clinical and oncologic characteristics were therefore examined in secondary or sensitivity analyses rather than forced into the primary model. Residual confounding by unmeasured or incompletely measured baseline characteristics cannot be excluded. Robustness was examined using conventional logistic regression, Firth penalized logistic regression [25], and cranial-reference CCR in all 73 patients. The relationship between CCR and the complete Frankel spectrum was examined with Spearman rank correlation as a supportive ordinal analysis.

AP and transverse preservation ratios, T2 signal, radial compression extent, circumferential compression, and number of compressed levels were examined in separate secondary imaging analyses. A secondary oncologic-context model included CCR, visceral metastatic disease, and hematologic malignancy. A solid-tumor-only restriction was evaluated as a sensitivity analysis. Detailed dimensional and secondary MRI morphology models are provided in Supplementary Table S3.

Among patients with baseline Frankel A-D deficit, early postoperative improvement was evaluated in relation to CCR, baseline Frankel grade, and deficit duration after log2 transformation. This longitudinal analysis was secondary and is summarized briefly in the main text; detailed transition and recovery-model results are provided in Supplementary Table S6; grade-level transitions are shown in Supplementary Figure S2.

No statistical imputation, stepwise variable selection, internally optimized ROC threshold, clustering procedure, or machine-learning classifier was used. Analyses were performed with available data, and analysis-specific denominators are reported. No formal adjustment for multiple comparisons was applied across secondary and exploratory analyses. These analyses were considered hypothesis-generating and were interpreted primarily according to effect estimates, confidence intervals, and consistency with the primary analysis rather than isolated *p* values. All tests were two-sided. A *p* value <0.05 was used as a conventional reference for the primary analysis. Analysis-specific missingness and denominators are summarized in Supplementary Table S5.

## 3. Results

### 3.1. Cohort Characteristics and Neurologic Presentation

Seventy-three patients constituted the final cohort after the exclusions shown in Figure 1. The median age was 65 years (IQR 59-71), and 24 patients (32.9%) were female. The preoperative Frankel distribution comprised 3 patients with grade A, 15 with grade B, 11 with grade C, 22 with grade D, and 22 with grade E. Thus, 22 of 73 patients (30.1%) were neurologically intact at presentation, whereas 51 (69.9%) had an objective neurologic deficit.

The most frequent primary tumor groups were lung cancer (20/73, 27.4%), multiple myeloma (12/73, 16.4%), breast cancer (7/73, 9.6%), gastrointestinal tumors (7/73, 9.6%), prostate cancer (6/73, 8.2%), renal cell carcinoma (6/73, 8.2%), lymphoma (5/73, 6.8%), and other solid tumors (10/73, 13.7%). Multiple myeloma was more frequent among Frankel-E patients (8/22, 36.4%) than among Frankel A-D patients (4/51, 7.8%). Hematologic malignancy was present in 17 patients overall.

Preoperative treatment exposure was similar between neurologic groups. Prior radiotherapy to any spinal site had been administered in 16 of 49 Frankel A–D patients with available data (32.7%) and 7 of 22 Frankel-E patients (31.8%; *p* = 1.000). Index-level radiotherapy had been delivered in 4 of 47 (8.5%) and 2 of 22 (9.1%), respectively (*p* = 1.000). Preoperative systemic anticancer therapy had been received by 17 of 48 Frankel A–D patients (35.4%) and 6 of 22 Frankel-E patients (27.3%; *p* = 0.590).

Bilsky grade 2 was present in 29 patients and grade 3 in 44. Frankel-E status was present in 13 of 29 patients with grade 2 disease (44.8%) and in 9 of 44 patients with grade 3 disease (20.5%). Grade 3 compression was therefore more common among Frankel A–D patients than among Frankel E patients (68.6% vs. 40.9%; *p* = 0.037). In a Bilsky-only modified Poisson model, grade 3 disease was associated with lower prevalence of Frankel-E status (PR 0.46, 95% CI 0.22–0.95; *p* = 0.035).

Mechanical disease did not show the same pattern. Median recalculated SINS was 10 (IQR 8–13) in patients with Frankel A-D deficit and 12.5 (IQR 9–14) in Frankel-E patients (*p* = 0.116). Pathologic fracture was present in 62.7% and 68.2%, respectively (*p* = 0.792), and kyphosis in 25.5% and 45.5% (*p* = 0.107). Selected baseline characteristics are shown in Table 1.

### 3.2. Quantitative Cord Deformation

The two-adjacent CCR was available in 71 patients: 50 with Frankel A–D deficit and 21 with Frankel-E status. Mean CCR was 0.430 ± 0.178 in patients with deficit and 0.561 ± 0.195 in neurologically intact patients. Median values were 0.439 (IQR 0.274–0.554) and 0.521 (IQR 0.467–0.650), respectively (*p* = 0.013; Figure 3A).

The absolute size of the reference cord did not differ meaningfully between neurologic groups. Median cranial reference area surrogate was 58.74 mm^2^ (IQR 50.73–67.09) in Frankel A–D patients and 55.77 mm^2^ (IQR 50.17–60.36) in Frankel-E patients (*p* = 0.424). Among the 71 patients with a caudal reference measurement, median caudal area was 53.43 mm^2^ (IQR 49.54–62.52) and 56.04 mm^2^ (IQR 48.19–61.99), respectively (*p* = 0.925). The group difference emerged at the compressed level: median maximal-compression area surrogate was 23.12 mm^2^ (IQR 17.05–32.32) in Frankel A–D patients and 32.01 mm^2^ (IQR 25.71–40.97) in Frankel-E patients (*p* = 0.013).

In the primary modified Poisson model, each 0.10 increase in two-adjacent CCR was associated with a 23% higher prevalence of Frankel-E status after accounting for Bilsky grade (PR 1.23, 95% CI 1.04–1.47; *p* = 0.018). In the same model, the estimate for Bilsky grade 3 attenuated to PR 0.63 (95% CI 0.30–1.33; *p* = 0.225). Conventional logistic regression produced a similar estimate for CCR (OR 1.40 per 0.10 increase, 95% CI 1.04–1.95; *p* = 0.035), and Firth penalized logistic regression yielded a similar estimate (OR 1.37, 95% CI 1.03–1.88; *p* = 0.033).

The full-cohort cranial-reference analysis was directionally consistent in all 73 patients. Each 0.10 increase in cranial-reference CCR was associated with a PR of 1.22 for Frankel-E status (95% CI 1.03–1.45; *p* = 0.019). The corresponding conventional logistic estimate was OR 1.37 (95% CI 1.04–1.86; *p* = 0.029), and the Firth estimate was OR 1.35 (95% CI 1.03–1.80; *p* = 0.028). Principal and sensitivity effect estimates are also displayed in Supplementary Figure S1. These analyses use substantially overlapping observations and should be regarded as robustness checks rather than independent replications.

Across the full ordinal Frankel scale, CCR showed a modest positive association with neurologic grade (Spearman rho = 0.329; *p* = 0.005; Figure 3B). The distributions were overlapping and did not increase monotonically at every adjacent grade, but the ordinal association provided supportive evidence that the primary Frankel E versus A–D finding was not solely an artifact of dichotomization.

Exploratory dimensional analysis suggested a somewhat clearer contribution from AP preservation than from transverse preservation. For the two-adjacent method, each 0.10 increase in AP preservation was associated with a PR of 1.23 for Frankel-E status (95% CI 1.02–1.49; *p* = 0.032), whereas the corresponding transverse estimate was PR 1.19 (95% CI 0.95–1.48; *p* = 0.136). These were secondary analyses and were not used to redefine the primary exposure.

Other static MRI descriptors were less informative. After adjustment for Bilsky grade, T2 hyperintensity was not associated with Frankel-E status (PR 0.79, 95% CI 0.39–1.61; *p* = 0.525). Neither increasing radial compression category (PR 0.92 per category, 95% CI 0.59–1.43; *p* = 0.695), circumferential compression (PR 0.75, 95% CI 0.30–1.88; *p* = 0.534), nor the number of compressed levels (PR 0.82 per additional level, 95% CI 0.46–1.45; *p* = 0.493) showed an independent association. Quantitative MRI findings are summarized in Table 2 and the principal regression estimates in Table 3. Detailed secondary imaging estimates are provided in Supplementary Table S3.

### 3.3. Interobserver Reliability Outcomes

Fifteen randomly selected patients underwent complete independent repeat quantitative assessment by the second observer, including independent selection of the maximal-compression slice. Agreement was strong for the individual dimensions measured at maximal compression. ICC(A,1) was 0.907 (95% CI 0.745–0.968) for AP diameter and 0.856 (95% CI 0.633–0.949) for transverse diameter. Reference measurements were generally similarly reproducible, although caudal transverse diameter showed greater variability, with ICC(A,1) = 0.693 (95% CI 0.287–0.886).

For the primary two-adjacent CCR, ICC(A,1) was 0.758 (95% CI 0.433–0.911; Figure 4A). The cranial-reference ratio had a lower point estimate of 0.629 (95% CI 0.213–0.856). The two-adjacent AP and transverse preservation ratios had ICCs of 0.847 and 0.846, respectively. Bland–Altman analysis of the primary CCR showed a mean Observer 2 minus Observer 1 difference of −0.033, with 95% limits of agreement from −0.233 to 0.167 (Figure 4B). Thus, there was little systematic directional bias, although the reliability sample was small, the confidence interval around the ICC was wide, and individual-level limits of agreement were not narrow. The complete reliability results are reported in Supplementary Table S2.

### 3.4. Oncologic Context

Visceral metastatic status was available in 70 patients. Visceral metastases were present in 36 of 48 Frankel A-D patients with available data (75.0%) and in 9 of 22 Frankel-E patients (40.9%; *p* = 0.008).

In a secondary model containing CCR, visceral metastatic disease, and hematologic malignancy, the association between CCR and Frankel-E status persisted (PR 1.21 per 0.10 increase, 95% CI 1.03–1.42; *p* = 0.020). Visceral metastasis showed a directionally adverse but imprecise association (PR 0.47, 95% CI 0.19–1.14; *p* = 0.095), whereas hematologic malignancy was not independently associated with neurologic status.

The full cohort included 56 solid-tumor patients. In the solid-tumor-only primary two-adjacent analysis, 55 patients had complete CCR data. Within this restricted analysis, the CCR estimate remained in the same direction but was less precise and crossed the conventional significance threshold (PR 1.18, 95% CI 0.95–1.47; *p* = 0.125). Bilsky grade 3 remained associated with lower Frankel-E prevalence (PR 0.31, 95% CI 0.10–0.91; *p* = 0.033). The cohort was too small for credible tumor-specific interaction testing, particularly across the individual histologic subgroups, so effect modification by tumor biology remains unresolved. Expanded oncologic-context, HALP, and solid-tumor sensitivity analyses are reported in Supplementary Table S4.

### 3.5. Early Postoperative Neurologic Course

Among the 51 patients who presented with Frankel A-D deficit, 22 (43.1%) improved by at least one Frankel grade by postoperative day 10–14. Across the full cohort, 9 of 29 patients in a baseline Frankel A-C category improved to a Frankel D-E category, whereas 1 of 44 patients in a baseline Frankel D-E category deteriorated to Frankel C.

CCR itself did not independently explain early postoperative improvement. In the complete deficit-duration model (*n* = 48), each doubling of preoperative motor-deficit duration was associated with a lower prevalence of early improvement (PR 0.81, 95% CI 0.68–0.95; *p* = 0.010), whereas CCR was not significant (PR 1.14 per 0.10 increase, 95% CI 0.94–1.38; *p* = 0.193). Detailed transition and recovery-model results are provided in Supplementary Table S6, and the complete Frankel-grade transition pattern is shown in Supplementary Figure S2.

## 4. Discussion

The main observation in this cohort is that measurable spinal cord deformation differed between neurologically intact and impaired patients even after the population had already been restricted to Bilsky grade 2–3 thoracic MESCC. Frankel-E patients retained greater normalized cord caliber at maximal compression, and this relationship persisted after Bilsky grade was included in the primary model. Modified Poisson, conventional logistic, Firth logistic, and cranial-reference analyses were generally consistent in direction, while the solid-tumor-only estimate was less precise and included the null. CCR also showed a modest positive ordinal association with Frankel grade, supporting the main result while emphasizing that the relationship is not a clean patient-level dose–response.

The result should not be read as an argument against the Bilsky classification. The ESCC scale was designed to provide a reproducible description of epidural disease [12], and it remains central to spine-oncology decision-making frameworks together with oncologic sensitivity, mechanical stability, and systemic fitness [3,4,5,26]. Bilsky grade retained clear neurologic relevance in the present cohort: 44.8% of grade 2 patients were Frankel E compared with 20.5% of grade 3 patients. The two measurements describe related but distinct aspects of anatomy. Bilsky grade describes the relationship among epidural tumor, cerebrospinal fluid, and the cord; CCR asks how much of the patient’s own cord caliber remains at the most deformed slice.

Our findings extend the work of Kim et al., who used a similar normalized AP x transverse measure in symptomatic surgical MESCC and found an association with postoperative ambulatory function [17]. Their study addressed prognosis after treatment. The present study addressed the state in which the patient arrives, including the subgroup that remains completely neurologically intact despite high-grade thoracic compression. This distinction is important because a measurement associated with baseline neurologic preservation is not necessarily a predictor of postoperative recovery. Consistent with that distinction, CCR was not an independent correlate of early Frankel improvement after a deficit had already developed.

The absolute measurements clarify what the ratio is capturing. Neurologically intact patients did not have larger reference cords; cranial and caudal reference area surrogates were similar between groups. The difference appeared at the lesion itself, where the Frankel-E group retained a larger area surrogate. This supports the interpretation that CCR principally reflects local deformation rather than constitutional cord size. We deliberately did not import the 0.84 cutoff reported by Kim et al. or derive a new threshold from the present data. With only 73 patients and 22 Frankel-E presentations, any internally optimized threshold would be unstable and likely optimistic.

The measurement remains imperfect. The reliability experiment was intentionally demanding because the second observer independently selected the maximal-compression slice before caliper placement. The primary CCR ICC of 0.758 is acceptable for exploratory group-level research, but the confidence interval was wide. More importantly, the median between-group CCR difference was approximately 0.08, whereas the Bland–Altman limits of agreement extended from −0.233 to 0.167. Thus, interobserver variation for an individual patient can exceed the average separation between the neurologic groups even when there is little systematic observer bias. This distinction is critical: CCR may be useful as a cohort-level quantitative phenotype, but the present manual implementation is not sufficiently precise for individual-patient classification, thresholding, or treatment allocation. Standardized axial acquisition, direct cord segmentation, and automated or semi-automated shape analysis would be more appropriate for future threshold development [20].

Other static MRI descriptors were less informative in this cohort. T2 hyperintensity, radial extent, circumferential compression, and the number of directly compressed levels were not independently associated with Frankel-E status after adjustment for Bilsky grade. These negative findings should not be interpreted as evidence that such features are biologically irrelevant. Intramedullary T2 signal is nonspecific and may reflect edema, venous congestion, ischemic change, gliosis, or myelomalacia. Likewise, the fraction of epidural circumference occupied by tumor is not equivalent to the deformation experienced by the cord. Prior imaging studies have reported variable relationships among T2 signal, ESCC severity, and functional outcomes [15,16,17,18,20].

Anatomy is not the whole explanation. Visceral metastases were more common among patients with neurologic deficit, and tumor histology was heterogeneous. Multiple myeloma was overrepresented among neurologically intact patients, whereas lymphoma and gastrointestinal tumors were seen only in the deficit group in this cohort. CCR retained a similar association when visceral disease and hematologic malignancy were added to a secondary model, but the solid-tumor-only estimate remained positive with a wider confidence interval that included the null. This may reflect reduced sample size, but it also emphasizes that neurologic injury from MESCC is a dynamic biological event. Growth velocity, vascularity, radiosensitivity, prior treatment, venous congestion, ischemia, and individual tissue tolerance may all modify the clinical effect of a given degree of static deformation. The present sample is too small for reliable histology-specific interaction testing, so whether tumor type modifies the strength of the CCR-neurologic association remains unresolved. Prior radiotherapy and systemic anticancer therapy were similar between neurologic groups, reducing concern about a major imbalance in these measured pretreatment exposures. However, treatment timing was heterogeneous, and corticosteroid dose and timing before MRI could not be reconstructed consistently. Residual confounding from pretreatment, including potential effects on edema, pain, or local tumor response, therefore remains possible.

Mechanical variables are similarly difficult to interpret in a surgical cohort. Frankel-E patients did not have lower SINS, fewer fractures, or less kyphosis. It would be incorrect to infer that mechanical disease is protective. Instability and mechanical pain are reasons to operate on a neurologically intact patient, while severe neurologic compression may lead to surgery in a patient with a different mechanical profile. Conditioning the entire cohort on surgery therefore creates substantial selection bias and may alter the apparent relationships among anatomy, systemic disease, and neurologic status. If nonsurgically managed patients were included, the cohort would contain a different mixture of neurologically intact patients selected for radiotherapy, mechanically stable lesions, radiosensitive tumors, and patients judged medically unsuitable for surgery. The present data cannot determine whether inclusion of those patients would attenuate or strengthen the association between CCR and neurologic status. Accordingly, both the 30.1% Frankel-E proportion and the observed CCR effect should be interpreted as characteristics of this surgically selected population rather than estimates directly generalizable to the full spectrum of high-grade MESCC.

The postoperative analysis was secondary but clinically coherent. Forty-three percent of patients with baseline deficit improved by at least one Frankel grade during the first two postoperative weeks. CCR did not independently explain this early improvement, whereas longer motor-deficit duration was associated with a lower likelihood of early recovery. The 10–14-day endpoint was deliberately chosen to characterize neurologic change close to the surgical episode, before later outcome becomes increasingly influenced by rehabilitation, postoperative radiotherapy, systemic therapy, tumor progression, and other post-discharge events. It therefore cannot capture delayed neurologic recovery or later deterioration, and the absence of an association between CCR and early improvement should not be extrapolated to longer-term neurologic outcome. These data cannot define an optimal surgical window or prove that operating a fixed number of hours earlier would have changed an individual outcome, but they are consistent with prior research linking baseline neurologic state and timing of treatment to functional recovery in metastatic cord compression [27,28,29].

The immediate clinical role of CCR should therefore remain modest. A relatively preserved ratio does not make high-grade compression benign, and a low ratio is not, by itself, an indication for surgery. The measurement should not replace neurologic examination, Bilsky grade, SINS, tumor biology, systemic condition, or multidisciplinary judgment. Its current value is more likely to be in research phenotyping and trial stratification. Modern metastatic-spine care increasingly combines high-dose conformal radiotherapy with selective decompression, separation surgery, and less invasive stabilization when appropriate [7,8,9,10,11]. At the same time, selected ambulatory patients—particularly some patients with Bilsky grade 2 disease and limited mechanical risk—may be considered for radiotherapy-based pathways rather than immediate decompression [30,31]. A continuous measure of actual cord deformation could refine such research questions, but the present surgical cohort cannot determine treatment allocation.

The study has several strengths: an anatomically restricted high-grade thoracic cohort, a patient-normalized quantitative imaging exposure, blinded measurement, an anatomically defined reference-level rule, similar reference cord size between neurologic groups, independent slice selection in the reliability experiment, and a deliberately parsimonious primary model without stepwise selection or optimized cutoffs. The limitations are equally important. The study is retrospective, single-center, and restricted to surgically treated patients. Selection into the cohort was influenced by neurologic deficit, instability, pain, tumor biology, systemic condition, referral patterns, and local practice, and the effect of including nonsurgical patients cannot be inferred from these data. The primary analysis is cross-sectional and cannot establish causality. The cohort is modest, with 22 Frankel-E patients overall and 21 in the primary two-adjacent analysis. MRI acquisition was not prospectively standardized, and the AP x transverse product is an area surrogate rather than direct segmentation. Only 15 patients were included in the reliability analysis, resulting in wide confidence intervals and limits of agreement that are broad relative to the between-group CCR difference. Histologies were heterogeneous, and the solid-tumor-only analysis was less precise. Preoperative radiotherapy and systemic therapy were described, but corticosteroid exposure could not be reconstructed consistently by dose and timing. The Frankel scale is also less granular than contemporary ISNCSCI/AIS assessment; it was used because it was consistently documented in the retrospective record, whereas complete ISNCSCI examinations were not available and retrospective conversion was considered unreliable. Deficit duration depended on retrospective documentation, and postoperative neurologic status at 10–14 days does not capture later rehabilitation, radiotherapy, systemic therapy, progression, or delayed recovery. Finally, no formal multiplicity correction was applied to the secondary and exploratory analyses, which should therefore be regarded as hypothesis-generating.

These limitations explain why the present finding should not be converted into a clinical rule. A prospective multicenter study should include surgical and nonsurgical patients, standardize MRI acquisition, use direct cord segmentation or validated automated shape analysis, prespecify reliability targets, record the tempo of neurologic decline, and validate any proposed prediction model externally before a quantitative threshold is used in clinical decision-making.

## 5. Conclusions

Among surgically treated patients with Bilsky grade 2–3 thoracic MESCC, neurologically intact patients retained greater normalized spinal cord caliber at maximal compression. The association between two-adjacent CCR and Frankel-E status persisted after accounting for Bilsky grade, and the principal logistic, Firth logistic, and cranial-reference sensitivity analyses were generally consistent in direction. The solid-tumor-only estimate also remained positive but was less precise and included the null. The difference was not explained by larger reference cord dimensions. Interobserver agreement was acceptable for exploratory group-level research but remains too imprecise for individual-patient thresholding.

CCR did not independently explain early postoperative neurologic recovery once a deficit had developed; deficit duration showed the clearer longitudinal association. Quantitative cord-caliber measurement may therefore complement conventional ESCC grading as a research phenotype of high-grade MESCC, but it should not yet be used as a stand-alone predictor or treatment threshold. Prospective external validation with standardized MRI and direct segmentation is warranted.

## Figures and Tables

**Figure 1:**
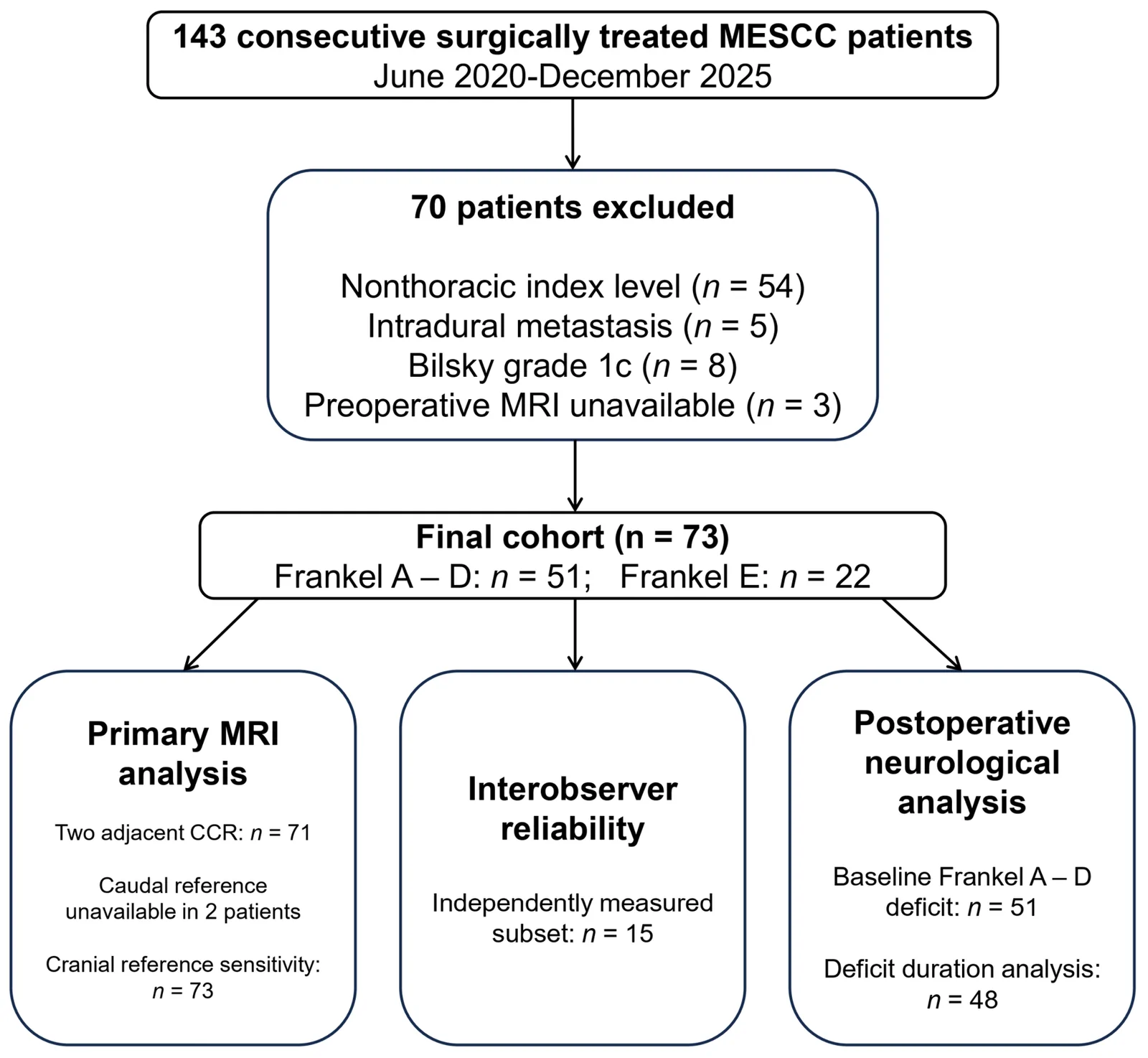
Study cohort derivation. Flow diagram showing selection from 143 consecutive surgically treated patients with spinal metastatic disease. Seventy patients were excluded, leaving 73 patients with thoracic Bilsky grade 2–3 MESCC. The two-adjacent CCR was available in 71 patients because a reliable caudal reference could not be obtained in two; both remained in the cohort and contributed to cranial-reference sensitivity analysis. Fifteen randomly selected patients underwent independent repeat measurement, and 51 patients with baseline Frankel A–D deficit contributed to the postoperative neurologic analysis.

**Figure 2:**
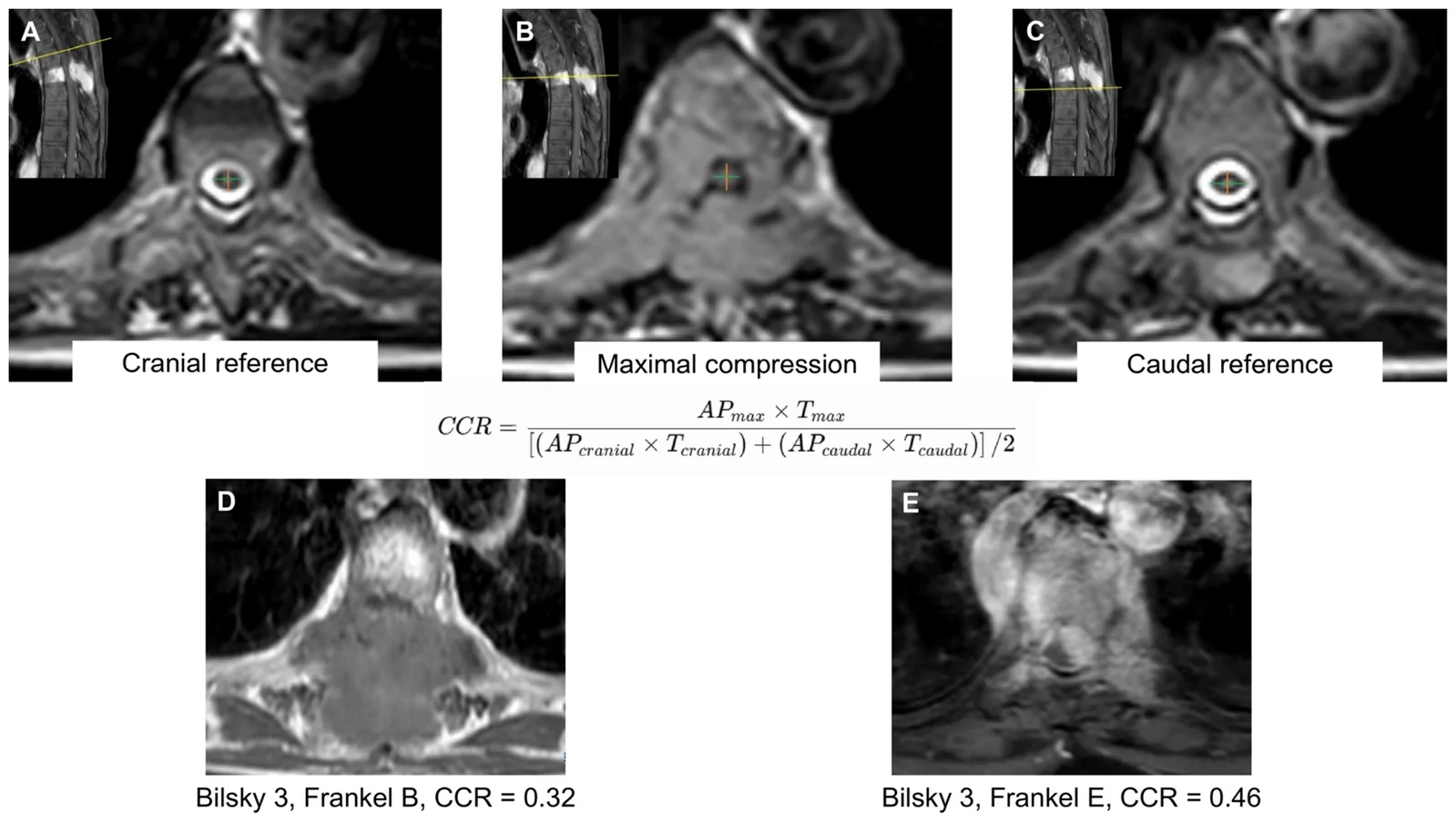
Quantitative spinal cord measurement method and representative neurologic–radiographic discordance. Axial T2-weighted MRI demonstrating the cranial reference level (**A**), maximal-compression level (**B**), and caudal reference level (**C**). AP and transverse spinal cord diameters were measured with electronic PACS calipers. Reference measurements were obtained at the first neighboring pedicle level in each direction at which no epidural mass was visible. The area surrogate was calculated as AP (orange line) × transverse (green line) diameter, and the two-adjacent CCR as the maximal-compression area surrogate divided by the mean of the cranial and caudal reference area surrogates. Panels (**D**) and (**E**) show two illustrative Bilsky grade 3 lesions with different quantitative deformation and neurologic presentations: Frankel B, CCR = 0.32; and Frankel E, CCR = 0.46, respectively.

**Figure 3:**
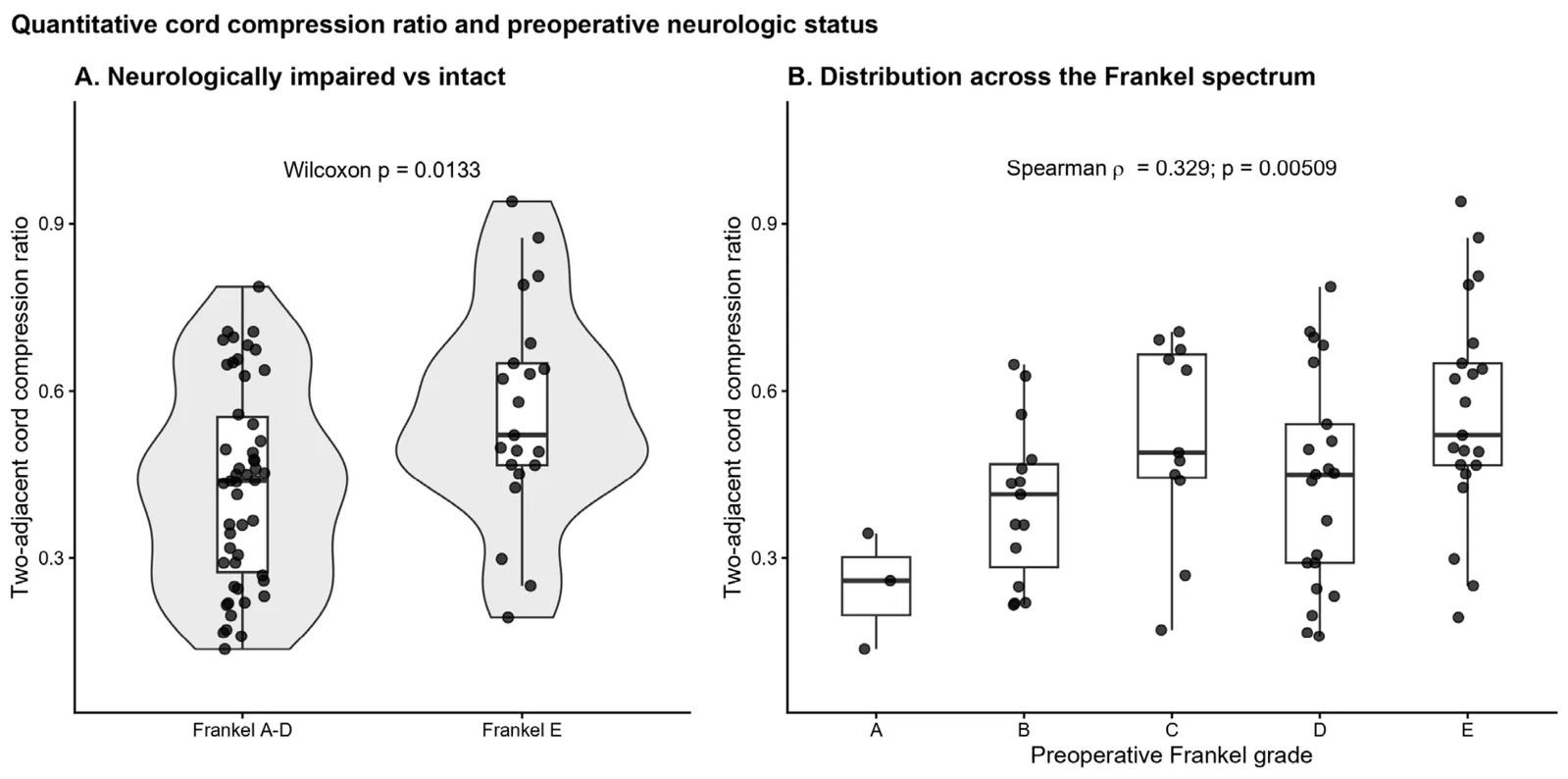
Quantitative cord compression ratio according to preoperative neurologic status. (**A**) Distribution of the two-adjacent CCR in patients presenting with Frankel A–D versus Frankel E neurologic status. Frankel-E patients had a higher median CCR (0.521 vs. 0.439; Wilcoxon *p* = 0.0133). (**B**) Distribution of CCR across the complete preoperative Frankel spectrum. Higher CCR values indicate greater preservation of normalized spinal cord caliber. The ordinal association was modest, and adjacent Frankel grades showed overlapping distributions (Spearman ρ = 0.329; *p* = 0.00509).

**Figure 4:**
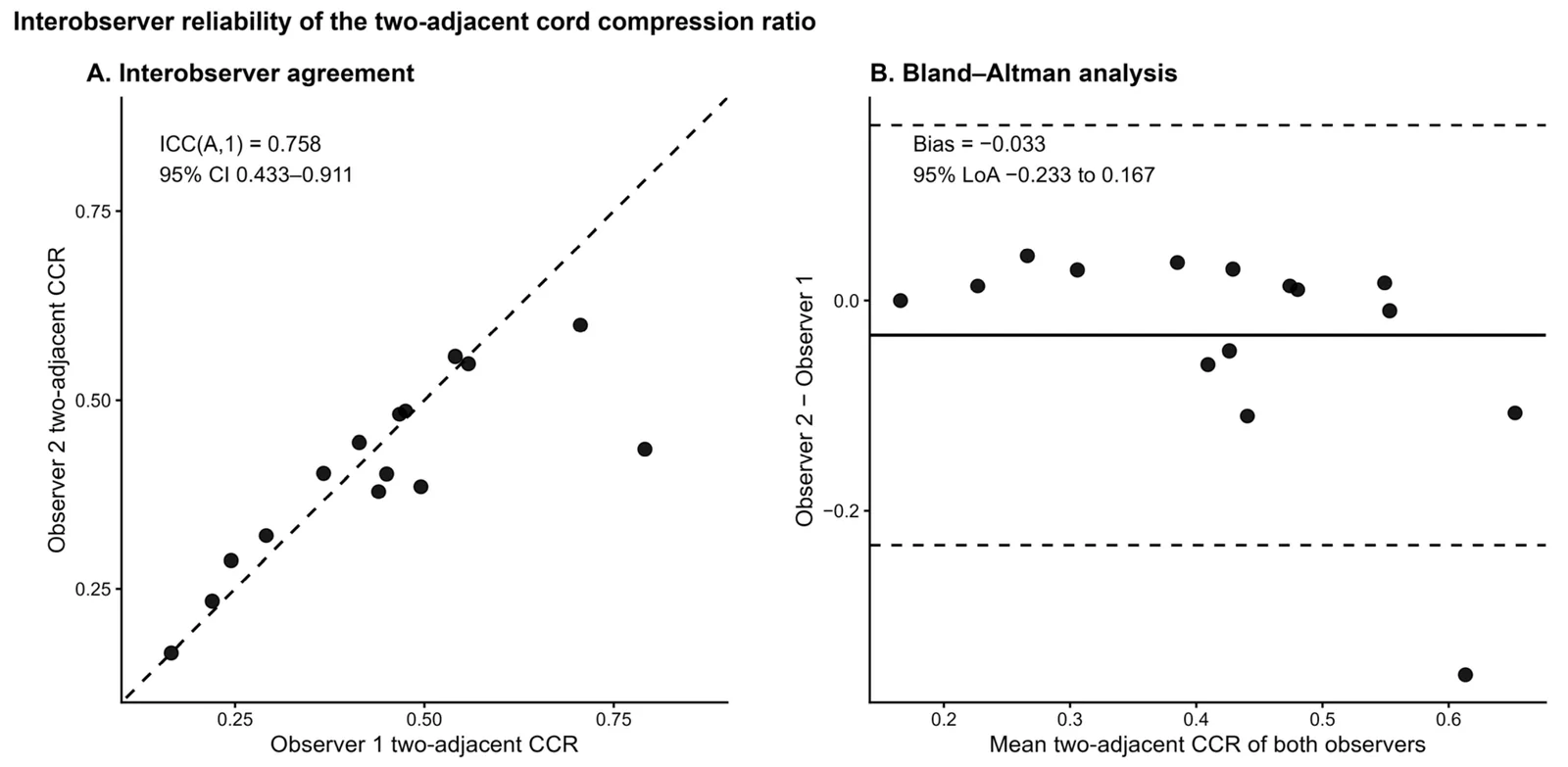
Interobserver reliability of the two-adjacent cord compression ratio. (**A**) Observer 1 and Observer 2 CCR measurements with the line of identity. The second observer independently selected the maximal-compression slice before measurement. ICC(A,1) = 0.758 (95% CI 0.433–0.911). (**B**) Corresponding Bland–Altman analysis. Mean Observer 2 minus Observer 1 bias was −0.033, with 95% limits of agreement from −0.233 to 0.167.

**Table 1:** Selected Baseline Characteristics According to Preoperative Neurologic Status.

Variable	Frankel A–D (*n* = 51)	Frankel E (*n* = 22)	*p* Value
Age, years, median (IQR)	65 (59–71)	63 (59.3–69.8)	0.459
Female sex	15 (29.4%)	9 (40.9%)	0.418
Primary tumor group	—	—	—
Lung	16 (31.4%)	4 (18.2%)	—
Multiple myeloma	4 (7.8%)	8 (36.4%)	—
Breast	4 (7.8%)	3 (13.6%)	—
Gastrointestinal	7 (13.7%)	0 (0.0%)	—
Prostate	6 (11.8%)	0 (0.0%)	—
Renal cell carcinoma	4 (7.8%)	2 (9.1%)	—
Lymphoma	5 (9.8%)	0 (0.0%)	—
Other solid tumors	5 (9.8%)	5 (22.7%)	—
Hematologic malignancy	9 (17.6%)	8 (36.4%)	0.129
Visceral metastasis *	36/48 (75.0%)	9/22 (40.9%)	0.008
Prior radiotherapy, any spinal site †	16/49 (32.7%)	7/22 (31.8%)	1.000
Prior index-level radiotherapy †	4/47 (8.5%)	2/22 (9.1%)	1.000
Preoperative systemic anticancer therapy ‡	17/48 (35.4%)	6/22 (27.3%)	0.590
Bilsky grade 3	35 (68.6%)	9 (40.9%)	0.037
Recalculated SINS, median (IQR)	10 (8–13)	12.5 (9–14)	0.116
Pathologic fracture	32 (62.7%)	15 (68.2%)	0.792
Kyphosis	13 (25.5%)	10 (45.5%)	0.107
T2 hyperintensity	31 (60.8%)	11 (50.0%)	0.445
Multilevel direct cord compression	28 (54.9%)	9 (40.9%)	0.315

Values are median (IQR) or *n* (%). Primary tumor subgroup rows are descriptive and no overall subtype-distribution *p* value is presented in this table. * Visceral metastatic status was unavailable in three patients. † Prior radiotherapy data were available in 71 patients for any spinal-site radiotherapy and in 69 patients for index-level radiotherapy. ‡ Preoperative systemic anticancer therapy status was available in 70 patients. Treatment-exposure *p* values are from Fisher’s exact tests. Abbreviations: IQR, interquartile range; SINS, Spinal Instability Neoplastic Score.

**Table 2:** Quantitative MRI Characteristics According to Preoperative Neurologic Status.

Variable	Frankel A–D	Frankel E	*p* Value
Two-adjacent CCR available, *n*	50	21	—
Two-adjacent CCR, mean ± SD	0.430 ± 0.178	0.561 ± 0.195	—
Two-adjacent CCR, median (IQR)	0.439 (0.274–0.554)	0.521 (0.467–0.650)	0.013
Cranial-reference CCR, median (IQR)	0.409 (0.272–0.566)	0.539 (0.446–0.740)	0.006
Cranial reference area surrogate, mm^2^	58.74 (50.73–67.09)	55.77 (50.17–60.36)	0.424
Caudal reference area surrogate, mm^2^ *	53.43 (49.54–62.52)	56.04 (48.19–61.99)	0.925
Maximal-compression area surrogate, mm^2^	23.12 (17.05–32.32)	32.01 (25.71–40.97)	0.013
Two-adjacent AP preservation	0.661 (0.515–0.763)	0.744 (0.643–0.842)	0.033
Two-adjacent transverse preservation	0.622 (0.541–0.777)	0.704 (0.648–0.866)	0.035

Values are median (IQR) unless otherwise specified. Group-comparison *p* values are from Wilcoxon rank-sum tests. * Available in 71 patients. Abbreviations: AP, anteroposterior; CCR, cord compression ratio; IQR, interquartile range; SD, standard deviation.

**Table 3:** Primary Association Between Quantitative Cord Deformation and Frankel-E Status.

Analysis	*n*	Variable	Effect Estimate	95% CI	*p* Value
Bilsky-only modified Poisson	73	Bilsky grade 3 vs. 2	PR 0.46	0.22–0.95	0.035
Primary modified Poisson	71	CCR per 0.10 increase	PR 1.23	1.04–1.47	0.018
Primary modified Poisson	71	Bilsky grade 3 vs. 2	PR 0.63	0.30–1.33	0.225
Conventional logistic sensitivity	71	CCR per 0.10 increase	OR 1.40	1.04–1.95	0.035
Firth logistic sensitivity	71	CCR per 0.10 increase	OR 1.37	1.03–1.88	0.033
Cranial-reference modified Poisson	73	CCR per 0.10 increase	PR 1.22	1.03–1.45	0.019
Cranial-reference logistic sensitivity	73	CCR per 0.10 increase	OR 1.37	1.04–1.86	0.029
Cranial-reference Firth sensitivity	73	CCR per 0.10 increase	OR 1.35	1.03–1.80	0.028

The primary modified Poisson model used HC3 heteroscedasticity-consistent robust covariance estimates. Abbreviations: CCR, cord compression ratio; CI, confidence interval; OR, odds ratio; PR, prevalence ratio.

## Data Availability

The deidentified data supporting the findings of this study are available from the corresponding author upon reasonable request, subject to institutional and ethical restrictions.

## Supplementary Materials

The following supporting information can be downloaded at: https://www.mdpi.com/article/10.3390/jcm15197702/s1, Table S1: Complete demographic, clinical, oncologic, laboratory, mechanical, imaging, and operative characteristics according to preoperative neurologic phenotype; Table S2: Interobserver Agreement for Quantitative Spinal Cord Measurements; Table S3: Exploratory Dimensional-Preservation and Secondary MRI Morphology Models for Frankel-E Status; Table S4: Oncologic-context, HALP, and solid-tumor sensitivity analyses; Table S5: Missing-data profile and analysis-specific denominators; Table S6: Early postoperative Frankel-grade transitions and recovery-model results; Figure S1: Forest plot of principal and sensitivity estimates for the relationship between CCR and neurologic status; Figure S2: Preoperative-to-postoperative Frankel-grade transitions.
