# Supplementary material for: Quantitative Spinal Cord Deformation Is Associated with Neurologic Status in High-Grade Thoracic Metastatic Epidural Spinal Cord Compression: A Retrospective Surgical Cohort
Source: J Clin Med. 2026 Oct 5;15(19):7702. doi: 10.3390/jcm15197702 (PMC13665715; doi:10.3390/jcm15197702)

Supplementary Tables

Table S1. Complete Demographic, Clinical, Oncologic, Laboratory, Mechanical, Imaging, and Operative Characteristics According to Preoperative Neurologic Phenotype

Variable	Frankel A-D (*n* = 51)	Frankel E (*n* = 22)	p Value
**Demographic and clinical characteristics**			
Age, years	65 (59–71)	63 (59.2–69.8)	0.459
Female sex	15 (29.4%)	9 (40.9%)	0.418
BMI, kg/m²	24.5 (22.7–29)	24.5 (23.5–30.8)	0.274
Symptom duration, days	35 (15–71)	53.5 (28.5–90.8)	0.182
ASA grade ≥3	42 (82.4%)	11 (50.0%)	0.009
Emergency ASA status	22 (43.1%)	1 (4.5%)	<0.001
ECOG ≥3	35 (68.6%)	0 (0.0%)	<0.001
MSTFI	2 (1–3)	1 (1–2)	0.009
**Oncologic and pretreatment characteristics**			
**Primary tumor group**	—	—	—
Lung	16 (31.4%)	4 (18.2%)	—
Multiple myeloma	4 (7.8%)	8 (36.4%)	—
Breast	4 (7.8%)	3 (13.6%)	—
Gastrointestinal	7 (13.7%)	0 (0.0%)	—
Prostate	6 (11.8%)	0 (0.0%)	—
Renal cell carcinoma	4 (7.8%)	2 (9.1%)	—
Lymphoma	5 (9.8%)	0 (0.0%)	—
Other solid tumors	5 (9.8%)	5 (22.7%)	—
Hematologic malignancy	9 (17.6%)	8 (36.4%)	0.129
Visceral metastasis	36/48 (75.0%)	9 (40.9%)	0.008
Primary tumor known preoperatively	26 (51.0%)	10 (45.5%)	0.800
Prior index-level radiotherapy	4/47 (8.5%)	2 (9.1%)	1.000
Prior radiotherapy, any spinal site	16/49 (32.7%)	7 (31.8%)	1.000
Preoperative systemic anticancer therapy	17/48 (35.4%)	6 (27.3%)	0.590
**Laboratory and systemic-context variables**			
Hemoglobin, g/dL	11.7 (9.9–12.7)	12.9 (10.7–14.6)	0.063
WBC, ×10⁹/L	9.08 (6.39–11.62)	8.96 (6.48–10.92)	0.759
Platelets, ×10⁹/L	270 (206–336)	264 (231–292)	0.995
Neutrophils, ×10⁹/L	6.7 (4.7–8.99)	5.37 (4.6–7.37)	0.239

Variable	Frankel A-D (*n* = 51)	Frankel E (*n* = 22)	p Value
Monocytes, $\times 10^9$/L	0.72 (0.53–0.92)	0.66 (0.49–0.81)	0.330
Lymphocytes, $\times 10^9$/L	1.25 (0.86–1.71)	1.67 (1.21–2.59)	0.024
Albumin, g/L	34 (29–39)	38.5 (35.2–40.8)	0.007
NLR	5.03 (3.7–8.92)	2.81 (1.88–4.72)	0.005
MLR	0.52 (0.37–0.79)	0.36 (0.26–0.48)	0.009
PLR	194.8 (142.4–302)	139.6 (101.9–189.1)	0.014
SII	1208.8 (881.9–2386.1)	708.1 (491–1218.1)	0.009
HALP	19.2 (10.6–28.8)	35.6 (23.1–53.5)	<0.001
**Mechanical and MRI descriptors**			
Bilsky grade 3	35 (68.6%)	9 (40.9%)	0.037
Recalculated SINS	10 (8–13)	12.5 (9–14)	0.116
Pathologic fracture	32 (62.7%)	15 (68.2%)	0.792
Kyphosis	13 (25.5%)	10 (45.5%)	0.107
T2 hyperintensity	31 (60.8%)	11 (50.0%)	0.445
Multilevel direct cord compression	28 (54.9%)	9 (40.9%)	0.315
Number of directly compressed levels	2 (1–2)	1 (1–2)	0.222
Circumferential dominant compression	23 (45.1%)	6 (27.3%)	0.197
**Operative characteristics**			
Operative time, min	230 (150–270)	342.5 (196.2–423.8)	0.002
Decompression-only procedure	16 (31.4%)	5 (22.7%)	0.577
Instrumented procedure	34 (66.7%)	17 (77.3%)	0.418
Corpectomy-containing procedure	4 (7.8%)	6 (27.3%)	0.057

Continuous variables are presented as median (IQR) and compared using Wilcoxon rank-sum tests; categorical variables are n (%) and compared using Fisher's exact test. Where data were missing, the available denominator is shown in the cell. Primary tumor subgroup rows are descriptive and no overall subtype-distribution p value is presented. P values in this descriptive table were not adjusted for multiple comparisons. ASA and ECOG are reported descriptively and were not used as explanatory covariates in the primary neurologic model; operative variables reflect treatment selected after presentation. Preoperative corticosteroid exposure was not tabulated because dose and timing before MRI could not be reconstructed consistently across the retrospective study period. Abbreviations: ASA, American Society of Anesthesiologists; BMI, body mass index; ECOG, Eastern Cooperative Oncology Group; HALP, hemoglobin-albumin-lymphocyte-platelet index (reported using hemoglobin converted from g/dL to g/L); MLR, monocyte-to-lymphocyte ratio; MSTFI, Metastatic Spinal Tumor Frailty Index; NLR, neutrophil-to-lymphocyte ratio; PLR, platelet-to-lymphocyte ratio; SII, systemic immune-inflammation index; SINS, Spinal Instability Neoplastic Score; WBC, white blood cell count.

Table S2. Interobserver Agreement for Quantitative Spinal Cord Measurements

Measurement	*n*	ICC(A,1)	95% CI	Bias*	95% Limits of Agreement
AP diameter, cranial reference, mm	15	0.822	0.510–0.939	0.211	-0.521 to 0.943
Transverse diameter, cranial reference, mm	15	0.848	0.613–0.946	0.079	-0.636 to 0.795
AP diameter, caudal reference, mm	15	0.908	0.753–0.968	0.111	-0.471 to 0.692
Transverse diameter, caudal reference, mm	15	0.693	0.287–0.886	-0.004	-0.848 to 0.840
AP diameter, maximal compression, mm	15	0.907	0.745–0.968	-0.034	-1.037 to 0.969
Transverse diameter, maximal compression, mm	15	0.856	0.633–0.949	-0.178	-1.187 to 0.831
Two-adjacent CCR	15	0.758	0.433–0.911	-0.033	-0.233 to 0.167
Cranial-reference CCR	15	0.629	0.213–0.856	-0.044	-0.305 to 0.217
Two-adjacent AP preservation ratio	15	0.847	0.611–0.945	-0.019	-0.199 to 0.160
Two-adjacent transverse preservation ratio	15	0.846	0.608–0.945	-0.024	-0.144 to 0.097
Cranial-reference AP preservation ratio	15	0.792	0.498–0.924	-0.027	-0.234 to 0.180
Cranial-reference transverse preservation ratio	15	0.786	0.485–0.922	-0.029	-0.178 to 0.120

ICC(A,1) denotes a two-way, absolute-agreement, single-measure intraclass correlation coefficient. *Bias is Observer 2 minus Observer 1. The second observer independently selected the maximal-compression slice before performing measurements. Abbreviations: AP, anteroposterior; CCR, cord compression ratio; CI, confidence interval.

Table S3. Exploratory Dimensional-Preservation and Secondary MRI Morphology Models for Frankel-E Status

Imaging Variable	*N*	Contrast	PR	95% CI	p Value
Two-adjacent AP preservation	71	per 0.10 increase	1.23	1.02–1.49	0.032
Two-adjacent transverse preservation	71	per 0.10 increase	1.19	0.95–1.48	0.136
Cranial-reference AP preservation	73	per 0.10 increase	1.24	1.01–1.51	0.037
Cranial-reference transverse preservation	73	per 0.10 increase	1.19	0.98–1.43	0.073
T2 hyperintensity	73	present vs. absent	0.79	0.39–1.61	0.525
Radial compression extent	73	per category increase	0.92	0.59–1.43	0.695
Circumferential dominant compression	73	present vs. absent	0.75	0.30–1.88	0.534
Number of directly compressed levels	73	per additional level	0.82	0.46–1.45	0.493

Each variable was evaluated in a separate modified Poisson regression adjusted for Bilsky grade (3 vs. 2), using HC3 heteroscedasticity-consistent robust covariance estimates. Dimensional preservation ratios are scaled per 0.10 increase. These analyses were exploratory and were not used to redefine the primary imaging exposure. No formal adjustment for multiple comparisons was applied; p values should therefore be interpreted as hypothesis-generating, with emphasis on effect estimates and confidence intervals. Abbreviations: AP, anteroposterior; CI, confidence interval; PR, prevalence ratio.

Table S4. Oncologic-Context, HALP, and Solid-Tumor Sensitivity Analyses

Analysis	*N*	Variable or Comparison	Estimate / Summary	95% CI	p Value
Oncologic-context modified Poisson	68	CCR per 0.10 increase	PR 1.21	1.03–1.42	0.020
Oncologic-context modified Poisson	68	Visceral metastasis, present vs. absent	PR 0.47	0.19–1.14	0.095
Oncologic-context modified Poisson	68	Hematologic malignancy, yes vs. no	PR 0.93	0.43–2.01	0.848
HALP phenotype comparison	73	Frankel A-D vs. Frankel E median (IQR)	19.2 (10.6–28.8) vs. 35.6 (23.1–53.5)	—	<0.001
Solid-tumor-only modified Poisson	55	CCR per 0.10 increase	PR 1.18	0.95–1.47	0.125
Solid-tumor-only modified Poisson	55	Bilsky grade 3 vs. 2	PR 0.31	0.10–0.91	0.033

The oncologic-context model included patients with available two-adjacent CCR and visceral-metastasis data (n = 68). Hematologic malignancy comprised myeloma and lymphoma. HALP was retained as an exploratory systemic-context comparison and was not entered into the primary neurologic model; for reporting, hemoglobin was converted from g/dL to g/L before calculating the standard HALP scale. The solid-tumor sensitivity analysis excluded hematologic malignancies and adjusted CCR for Bilsky grade. Modified Poisson models used HC3 robust covariance estimates. No formal adjustment for multiple comparisons was applied to these secondary and exploratory analyses; corresponding p values should be interpreted cautiously and together with effect estimates and confidence intervals. Abbreviations: CCR, cord compression ratio; CI, confidence interval; HALP, hemoglobin-albumin-lymphocyte-platelet index (reported using hemoglobin converted from g/dL to g/L); IQR, interquartile range; PR, prevalence ratio.

Table S5. Missing-Data Profile and Analysis-Specific Denominators

Variable or Analysis	Available N	Missing N	Comment
Core demographics, preoperative Frankel grade, Bilsky grade, SINS, T2 signal, radial extent, dominant direction, compression-level count	73	0	Complete in final cohort
Visceral metastatic status	70	3	Used only when available; no imputation
BMI	67	6	Descriptive only
Prior index-level radiotherapy	69	4	Descriptive only
Prior radiotherapy, any spinal site	71	2	Descriptive only
Preoperative systemic anticancer therapy	70	3	Descriptive only
Preoperative corticosteroid exposure	—	—	Not analyzed; dose and timing before MRI could not be reconstructed consistently across the retrospective study period
Operative time	65	8	Descriptive only
Laboratory variables used in Table S1	73	0	No laboratory variable was an eligibility criterion
Cranial reference dimensions and cranial-reference CCR	73	0	Full-cohort sensitivity measure
Caudal reference dimensions	71	2	Reliable caudal cord reference unavailable in two patients
Two-adjacent CCR and two-adjacent dimensional preservation ratios	71	2	Primary quantitative MRI denominator
Primary modified Poisson / logistic / Firth analyses	71	2	Two-adjacent CCR + Bilsky grade
Cranial-reference sensitivity analyses	73	0	All final-cohort patients
Oncologic-context model	68	5	Complete cases for two-adjacent CCR, visceral metastasis, and hematologic status
Solid-tumor-only sensitivity analysis	55	—	Restricted analysis after excluding 17 hematologic malignancies; one additional two-adjacent CCR unavailable
Interobserver reliability analysis	15	—	Randomly selected independently remeasured subset; not missing data
Baseline Frankel A-D postoperative-improvement analysis	51	—	Restricted by design to patients with objective baseline deficit
Deficit-duration recovery model	48	3 of 51	Deficit onset could not be established reliably in three baseline-deficit patients; no imputation

No statistical imputation was performed. Missingness therefore affected analysis-specific denominators only. The three patients without preoperative MRI were excluded during cohort derivation, before entry into the final n = 73 cohort, as shown in Figure 1. Preoperative corticosteroid exposure is listed separately because the limitation was not simple missingness: dose and timing before MRI could not be reconstructed consistently enough for a reliable retrospective variable.

Table S6. Early Postoperative Frankel-Grade Transitions and Recovery-Model Results

Outcome or Model Variable	Result / Effect Estimate	95% CI	p Value
**A. Descriptive early neurologic course**			
Baseline Frankel A-D	51		
Improved by ≥1 Frankel grade by postoperative day 10–14	22/51 (43.1%)		
Baseline Frankel A-C, full cohort	29		
Transitioned to Frankel D-E by postoperative day 10–14	9/29 (31.0%)		
Remained Frankel A-C	20/29 (69.0%)		
Baseline Frankel D-E, full cohort	44		
Remained Frankel D-E	43/44 (97.7%)		
Deteriorated to Frankel C	1/44 (2.3%)		
Exact paired Frankel A-C versus D-E transition test			0.021
**B. Modified Poisson model for early Frankel improvement (n = 50)**			
CCR per 0.10 increase, adjusted for baseline Frankel grade	PR 1.10	0.91–1.32	0.336
**C. Modified Poisson model including deficit duration (n = 48)**			
CCR per 0.10 increase	PR 1.14	0.94–1.38	0.193
Baseline Frankel grade	PR 0.93	0.66–1.30	0.657
Deficit duration per doubling	PR 0.81	0.68–0.95	0.010
**D. Firth logistic sensitivity analysis (n = 48)**			
Deficit duration per doubling	OR 0.67	0.40–1.02	0.059

Postoperative neurologic status was assessed on postoperative day 10–14. Improvement was defined as an increase of at least one Frankel grade. Frankel D-E and Frankel A-C were used only as Frankel-based neurologic categories and should not be interpreted as separately observed walking status. The n = 50 model included baseline-deficit patients with an available two-adjacent CCR and adjusted for baseline Frankel grade. Reliable deficit-duration data were available for 48 patients; no imputation was performed. The Firth model was used as a sensitivity analysis. Abbreviations: CCR, cord compression ratio; CI, confidence interval; OR, odds ratio; PR, prevalence ratio.

Figure S1: Forest plot of principal and sensitivity estimates for the relationship between CCR and neurologic status

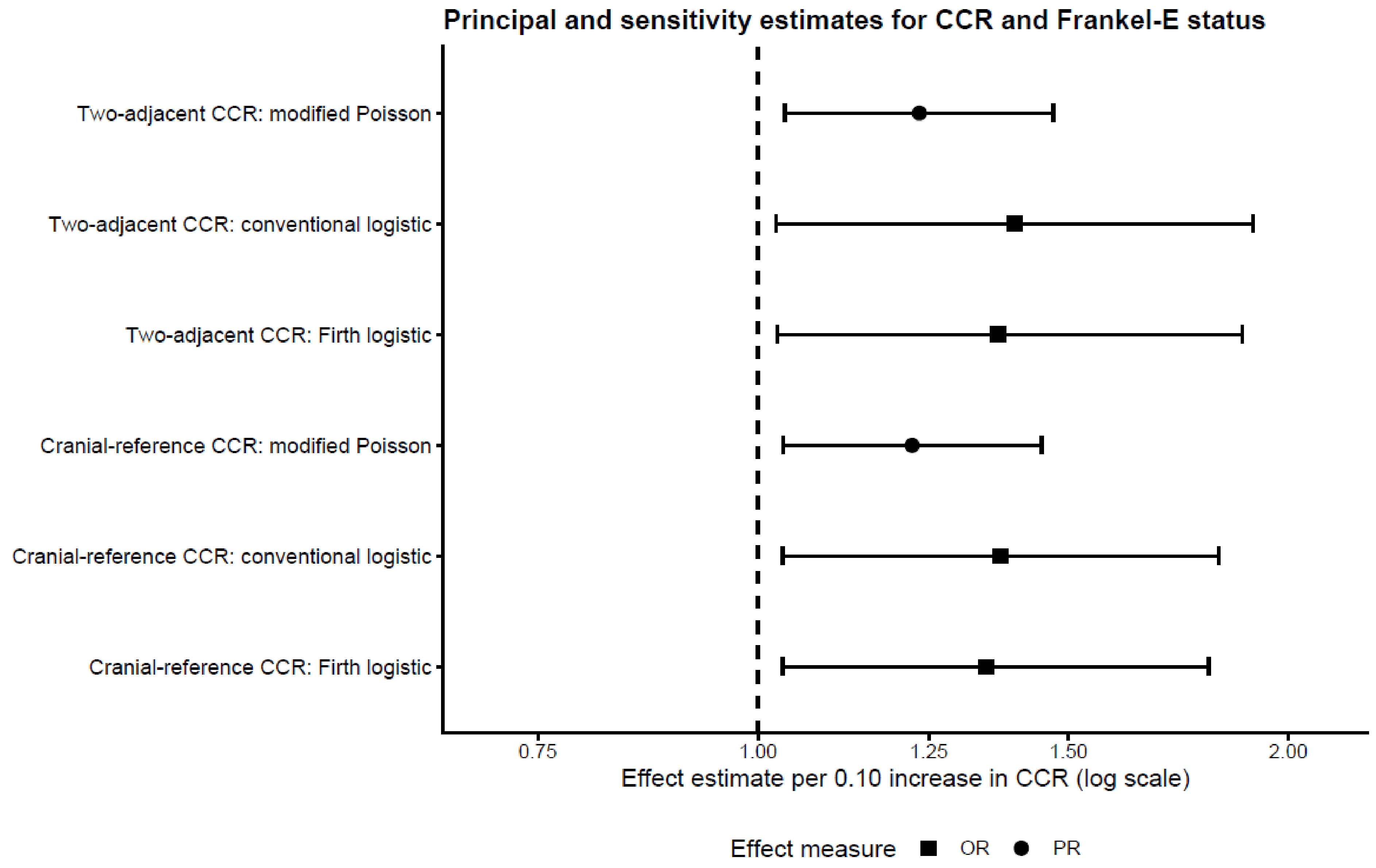

Figure S2: Preoperative-to-postoperative Frankel-grade transitions

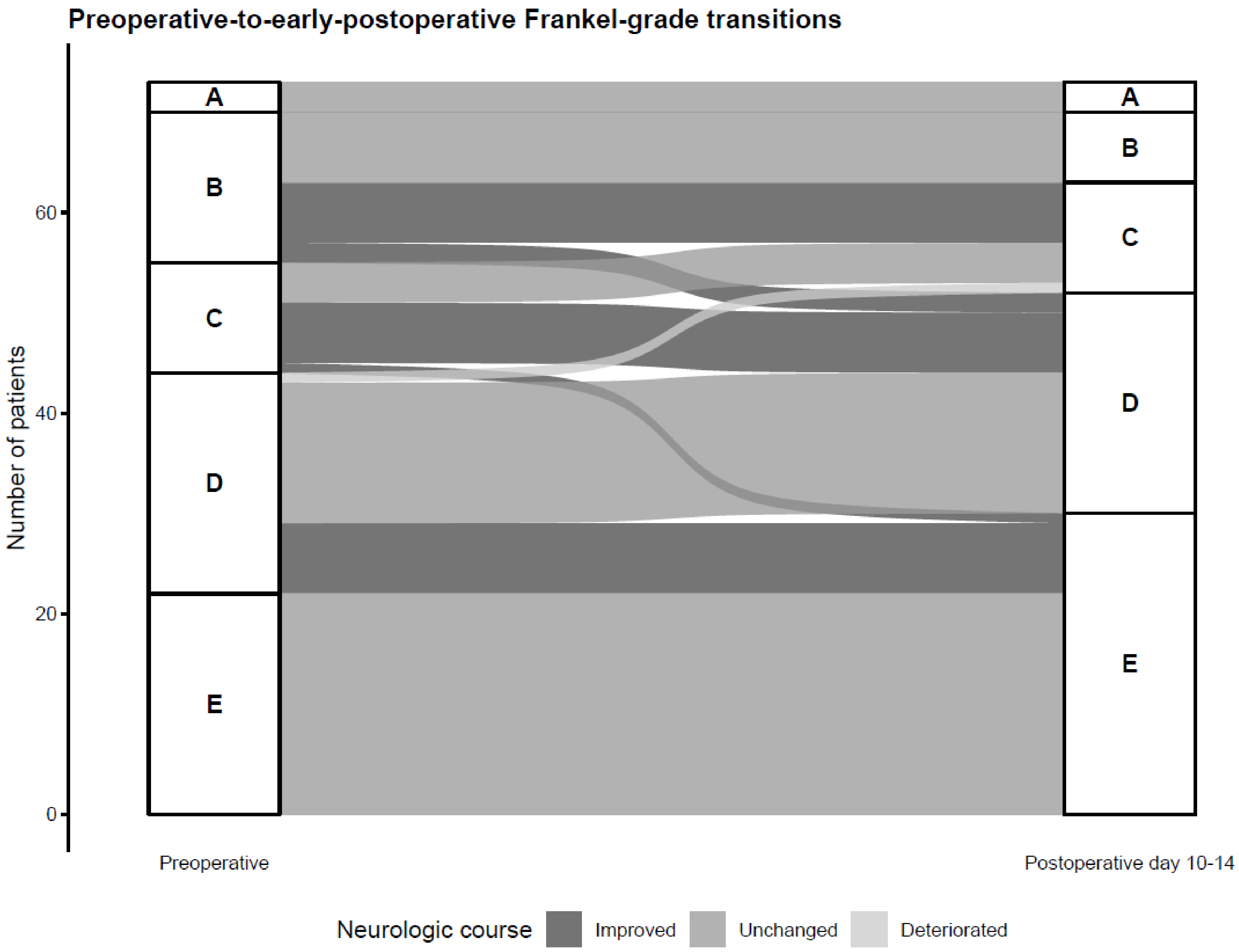

Supplement: Supplementary file 1 [file jcm-15-07702-s001.zip › jcm-4551775-supplementary.pdf]
